# Integrated Quantitative Transcriptome Maps of Human Trisomy 21 Tissues and Cells

**DOI:** 10.3389/fgene.2018.00125

**Published:** 2018-04-24

**Authors:** Maria Chiara Pelleri, Chiara Cattani, Lorenza Vitale, Francesca Antonaros, Pierluigi Strippoli, Chiara Locatelli, Guido Cocchi, Allison Piovesan, Maria Caracausi

**Affiliations:** ^1^Department of Experimental, Diagnostic and Specialty Medicine, Unit of Histology, Embryology and Applied Biology, University of Bologna, Bologna, Italy; ^2^Neonatology Unit, Sant’Orsola-Malpighi Polyclinic, Bologna, Italy; ^3^Neonatology Unit, Sant’Orsola-Malpighi Polyclinic, Department of Medical and Surgical Sciences, University of Bologna, Bologna, Italy

**Keywords:** integrated transcriptome map, meta-analysis, human chromosome 21, trisomy 21, Down syndrome

## Abstract

Down syndrome (DS) is due to the presence of an extra full or partial chromosome 21 (Hsa21). The identification of genes contributing to DS pathogenesis could be the key to any rational therapy of the associated intellectual disability. We aim at generating quantitative transcriptome maps in DS integrating all gene expression profile datasets available for any cell type or tissue, to obtain a complete model of the transcriptome in terms of both expression values for each gene and segmental trend of gene expression along each chromosome. We used the TRAM (Transcriptome Mapper) software for this meta-analysis, comparing transcript expression levels and profiles between DS and normal brain, lymphoblastoid cell lines, blood cells, fibroblasts, thymus and induced pluripotent stem cells, respectively. TRAM combined, normalized, and integrated datasets from different sources and across diverse experimental platforms. The main output was a linear expression value that may be used as a reference for each of up to 37,181 mapped transcripts analyzed, related to both known genes and expression sequence tag (EST) clusters. An independent example *in vitro* validation of fibroblast transcriptome map data was performed through “Real-Time” reverse transcription polymerase chain reaction showing an excellent correlation coefficient (*r* = 0.93, *p* < 0.0001) with data obtained *in silico*. The availability of linear expression values for each gene allowed the testing of the gene dosage hypothesis of the expected 3:2 DS/normal ratio for Hsa21 as well as other human genes in DS, in addition to listing genes differentially expressed with statistical significance. Although a fraction of Hsa21 genes escapes dosage effects, Hsa21 genes are selectively over-expressed in DS samples compared to genes from other chromosomes, reflecting a decisive role in the pathogenesis of the syndrome. Finally, the analysis of chromosomal segments reveals a high prevalence of Hsa21 over-expressed segments over the other genomic regions, suggesting, in particular, a specific region on Hsa21 that appears to be frequently over-expressed (21q22). Our complete datasets are released as a new framework to investigate transcription in DS for individual genes as well as chromosomal segments in different cell types and tissues.

## Introduction

Down syndrome (DS) is the first genetic alteration to have been described in humans (Lejeune et al., 1959), is the most frequent human chromosomal disorder and it causes mainly intellectual disability (ID). DS or trisomy 21 (T21) is characterized by the presence of an extra full or partial chromosome 21 (Hsa21), but the molecular mechanisms at the basis of the pathogenesis are still unclear. The identification of genes contributing to DS phenotype and its phenotypic variability is necessary to understand the DS pathogenesis and could be the key to any targeted therapeutic treatment (Gardiner et al., 2010).

Trisomy 21 results in the duplication of over 400 genes (Sturgeon et al., 2012). According to the simplest model of gene expression in DS, a 3:2 ratio for Hsa21 genes should be expected, but there is evidence of under-expressed Hsa21 genes and dysregulation of genes located on other chromosomes than Hsa21 (Letourneau et al., 2014).

Two different hypotheses have been proposed to explain DS phenotype: “developmental instability” and “gene-dosage effect” (Ait Yahya-Graison et al., 2007). According to the first hypothesis, the presence of an extra Hsa21 globally disturbs the correct balance of gene expression in DS cells during development (Saran et al., 2003) and determines a non-specific disturbance of genomic regulation and expression (Vilardell et al., 2011) resulting in a disruption of homeostasis throughout the genome. The second theory of the “gene dosage effect” states that the over-expression of duplicated genes on Hsa21 directly contributes to different aspects of DS phenotype (Korenberg, 1990). To determine which hypothesis applies to the etiology of DS, a number of investigators have conducted gene-expression studies in mouse models and human tissues or cell lines. Several methods have been used, including microarrays, serial analysis of gene expression (SAGE), Real-Time RT-PCR, RNA-seq or proteomic approaches (Lockstone et al., 2007; Prandini et al., 2007; Volk et al., 2013; Kong et al., 2014; Zhao et al., 2016; Liu et al., 2017).

However, the different studies show contrasting results, probably deriving from differences due to tissue specificity, developmental stages, as well as the applied experimental platforms and statistical techniques (Letourneau et al., 2014; Do et al., 2015; Sullivan et al., 2016), suggesting that the two hypotheses are not mutually exclusive and that the DS phenotype is probably caused by both mechanisms (Antonarakis, 2001).

Recently, another suggested mechanism which may affect global gene expression in trisomic cells is based on differences in chromatin topology that might generate gene expression dysregulation domains (GEDDs), i.e., genes clustered into large chromosomal domains of activation or repression (Letourneau et al., 2014). However, independent re-analysis of this RNA-seq dataset has questioned the validity of GEDDs in DS (Do et al., 2015). Therefore, an open issue is the identification of relevant gene expression changes caused by T21 and the characterization of variability across cell types, tissue types, genetic backgrounds, and developmental stages (Olmos-Serrano et al., 2016; Sullivan et al., 2016).

Several tools have been developed to perform analysis of gene expression profile datasets. We aim at generating quantitative transcriptome maps in DS, integrating all gene expression profile datasets available for each cell type or tissue, to obtain a complete model of the transcriptome in terms of both expression values for each gene and segmental trend of gene expression along each chromosome. TRAM (Transcriptome Mapper) (Lenzi et al., 2011) is a software able to integrate gene expression data from different sources and to provide quantitative transcriptome maps of specific cells or tissues. TRAM has been used in recent years to carry out analyses of gene expression (Caracausi et al., 2014, 2016, 2017b; Pelleri et al., 2014; Mariani et al., 2016; Rodia et al., 2016; Vitale et al., 2017b) since transcriptome maps can be easily generated, also showing differential expression between two biological conditions (e.g., pathological vs. normal). In particular, two key points of the original TRAM approach need to be underlined.

First, the data are “integrated”, thus generating a normalized, consensus linear value for the expression level of every gene represented in at least one of the platforms used in any study related to a given biological condition (e.g., a specific tissue). None of the original papers offers this type of numerical analysis of their raw data, each report being exclusively focused on its own data. We have previously repeatedly and consistently shown that the TRAM algorithm is able to effectively produce biologically meaningful results, based on a pipeline including uniformation and verification of different gene identifiers, followed by intra- as well as inter-sample normalization based on both parametric and non-parametric summarization of the data, plus a unique original method (“scaled quantiles”) able to circumvent the problem of integration of the data from microarray platforms representing gene sets of highly diverse numerosity (Lenzi et al., 2011; Piovesan et al., 2013; Caracausi et al., 2017a). Moreover, statistically highly significant correlation between *in silico* and *in vitro* data has repeatedly been obtained by Real-Time RT-PCR whenever human RNA from analogous biological conditions was available, proving the reliability and efficiency of TRAM software [(Caracausi et al., 2014): whole brain, cerebellum, cerebral cortex; (Caracausi et al., 2016): hippocampus; (Caracausi et al., 2017b): whole heart; (Vitale et al., 2017b): whole thyroid]. We also have shown (Lenzi et al., 2011; Vitale et al., 2017b) that increasing the sample size thanks to effective cross-platform data integration from different sources leads to a reduction of systematic bias associated with each different platform, thus generating a final consensus value for the mean expression level of that gene in a given tissue much more similar to the actual mean value, with results that outperform similar elaborations conducted in absence of the integration and normalization pipeline offered by TRAM at the cost of an initial manual curation of the datasets to be included in the meta-analysis (comparison reported in Vitale et al., 2017b).

In addition, while comparison of gene expression profiles typically generates lists of over-/under-expressed genes, there is actually no simple means to extract a consensus, reference gene expression numerical value for thousands of transcripts present in a homogeneous biological condition (e.g., a given normal tissue or a given trisomic tissue). For instance, it would be impossible to query these lists to readily identify both the quantitative expression value as well as the aneuploid/euploid ratio for any given gene (provided that it is represented in at least one experimental platform from which original data were derived).

The aim of this study is to build a systematic, quantitative framework of gene expression in DS, comparing different types of trisomy 21 and normal tissues. In the present work, we performed gene expression analyses, focusing on relationships between gene expression and map location as well as functional analysis of genes with an altered expression due to trisomy 21. In addition, the released database of gene expression in DS will allow to test hypotheses regarding specific mechanisms involved in DS pathogenesis.

## Materials and Methods

### Database Search and Selection

A systematic biomedical literature search was performed up to May 2016 in order to identify articles related to global gene expression profile experiments in DS patients. A general search using the expression “Down syndrome”[MeSH] AND (“Gene Expression Profiling”[MeSH] OR “Oligonucleotide Array Sequence Analysis”[MeSH] OR “Microarray Analysis”[MeSH] OR microarray^∗^ OR “Expression profile” OR SAGE) was performed in PubMed^1^.

Moreover, The NCBI GEO (National Center for Biotechnology Information-Gene Expression Omnibus) (Barrett and Edgar, 2006) functional genomic repository was searched for: “Down syndrome”[MeSH] AND “Homo sapiens”[Organism]. The EBI (European Bioinformatics Institute) ArrayExpress (Brooksbank et al., 2014) database of functional genomic experiments was searched at the website^2^ for the terms “Down syndrome”, “Trisomy 21”, choosing “Homo sapiens” as organism.

Search results were then filtered using inclusion and exclusion criteria. The inclusion criteria were availability of raw or pre-processed data and experiments performed on human DS vs. normal biosamples. The exclusion criteria were data derived from treated cells or tissues or arising from fetuses and embryonic annexes; experiments conducted on exon arrays (the processing of data by TRAM is impeded because of an extremely high number of data points); experiments on platforms whose probes are divided across multiple slides (hindering intra-sample normalization); lack of gene identifiers corresponding to those found in the records of GEO (GSM standards) or ArrayExpress; platforms that analyze an atypical number of genes (i.e., <5,000 or >60,000).

In order to obtain linear quantitative transcriptome maps, values from each dataset were linearized when provided as logarithms. When only raw files (e.g., File CEL) were available, they were pre-processed using the Alt Analyze software (Emig et al., 2010).

### TRAM (Transcriptome Mapper) Analysis

TRAM software is able to import gene expression data from GEO, ArrayExpress databases or in a custom source in tab-delimited text format whether the data are referred to microarray or RNA-seq platforms, for the creation and analysis of quantitative transcriptome maps (Lenzi et al., 2011).

We used an updated version of TRAM (TRAM 1.3^3^, set up with human gene data downloaded from NCBI up to November 11, 2017) including enhanced resolution of gene identifiers through updated NCBI Gene database, updated platform annotation files and UniGene data parsing (Lenzi et al., 2006).

Firstly, TRAM performs an intra-sample normalization by transforming each raw intensity value as percentage of the mean value in that sample, equivalent to the classic “global normalization” in the microarray data analysis (Quackenbush, 2002). Following this first round of normalization, inter-sample normalization (scaled quantile normalization) of gene expression values from multiple platforms is performed allowing robust comparison across experimental platforms even with a highly diverse numerosity of analyzed features (Lenzi et al., 2011). The value for each locus, in each biological condition, is represented by the mean value of all the values available for that locus. The mean value of gene expression of the whole genome is used to determine the percentile of expression for each gene. The comparison of two different biological conditions (Pool A and Pool B) allows the analysis of differential maps using the ratio of the mean expression values for each locus (A/B), in addition to the maps related to each single pool.

For the creation of the maps, TRAM software does not include the probes for which an expression value is not available, assuming that the level of expression was not measured; furthermore, raw expression values lower or equal to zero are thresholded by TRAM to 95% of the minimum positive value present in that sample. Choosing “0” as the expression value would create difficulty to adequately assess differential expression since the value “x/0” has no meaning, so TRAM sets values (≤0) to 95% of the minimum detected value to obtain defined numbers when it is necessary to calculate a ratio between the values of the Pool A and the Pool B and to capture very high over-expression which would be lost if choosing “0”. Assuming that in these cases the expression level is too low to be detected with the experimental conditions used, this transformation is useful to highlight a difference in gene expression.

**FIGURE 1:**
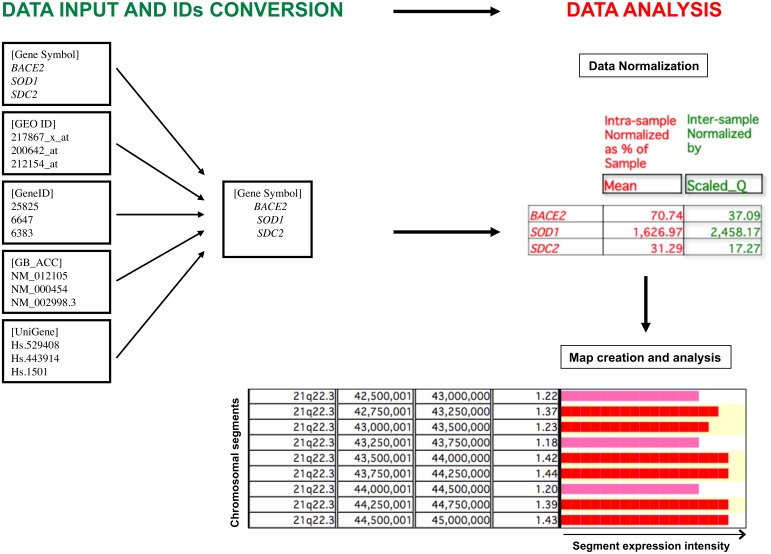
Graphic representation of the TRAM software workflow. The software allows the import and analysis of gene expression profile datasets in tab-delimited text format. Gene expression values are assigned to individual loci following conversion of all types of gene identifiers (IDs) into official gene symbols, and submitted to an intra- and inter-sample normalization. The value for each locus is the mean value of all available normalized values for that locus. The expression ratio obtained from the comparison of two different conditions is graphically displayed for each chromosomal segment, expressed as ratio of the mean of the expression values of the loci included in that segment. Over- and under-expressed regions are then determined following statistical analysis.

Finally, a graphical representation of the gene expression profile is created in two different modes, “Map” or “Cluster”, identifying critical genomic regions or genes (genomic regions including one gene) with significant differential expression comparing two different biological conditions. We focused mainly on the “Map” mode, analyzing over-/under-expressed segments of the genome, with a window size of 500,000 bp and a shift of 250,000 bp (default parameters). The expression value for each genomic segment is calculated by the mean of the expression values of the loci included in that segment. We did not consider loci for which mean value was derived from less than three biological samples. A segment is first tagged as over-/under-expressed using descriptive statistics if, considering the distribution of size and density of human genes (Piovesan et al., 2015, 2016), that segment contains at least three genes having an expression value within the highest and the lowest 2.5th percentile determined by the mean value of gene expression of the whole genome (default parameters) and if that segment has also a value of expression within the highest and the lowest 2.5th percentile among all genomic segments (default parameter). The statistical significance is then assessed by statistical tests based on hypergeometric distribution, a recognized algorithm able to test the probability ‘p’ that colocalization of three over-/under-expressed genes within the same chromosomal segment may be due to chance (Lenzi et al., 2011; Caracausi et al., 2016). The *p*-value is finally corrected for multiple comparisons possible causing False Discovery Rate (FDR) due to the high number of segments or genes in a genome. A segment or a gene was considered to be statistically significantly over- or under-expressed for *q* < 0.05. A graphical representation of the overall TRAM software workflow is provided in **Figure 1**.

Significance of the over-/under-expression for single genes can be determined by running TRAM in “Map” mode and lowering the segment window to 25,000 bp with a minimum number of over-/under-expressed genes in that window equal to 1. Since this window size is lower than 40% of the mean size of a human protein-coding gene which was determined to be 67 kb by searching the recent GeneBase database (Piovesan et al., 2016) (mean gene length calculated in 17,958 “reviewed” or “validated” entries available in the NCBI Gene April 2015 annotation release), the significant over-/under-expression of a segment almost always corresponds to that of the gene located in it. When the segment window contains more than one gene, the significance is maintained if the expression value of the over-/under-expressed gene prevails over the others.

In order to obtain quantitative framework of gene expression in DS and normal cells, we selected datasets related to several tissues and cell types from different origins: brain; lymphoblastoid cell lines (LCLs); blood cells; fibroblasts; thymus and induced pluripotent stem cells (iPSCs). For each one we created a directory (folder) for DS (Pool A) and normal (Pool B) condition. In addition, Pool A containing all DS samples for any tissue and Pool B containing all normal samples were created to generate a global view of DS transcriptome (“Total transcriptome map”). Experimental design scheme is shown in **Table 1**.

**Table 1:** Experimental design.

TRAM analysis		DS samples	Normal samples
A	TRAM DS brain vs. normal brain	13	11
B	TRAM DS LCLs vs. normal LCLs	17	18
C	TRAM DS blood vs. normal blood	6	5
D	TRAM DS fibroblasts vs. normal fibroblasts	11	14
E	TRAM DS thymus vs. normal thymus	4	4
F	TRAM DS iPSCs vs. normal iPSCs	32	22
G	Whole pool DS vs. whole pool normal (“Total transcriptome map”)	83	74

*We performed analysis for each cell/tissue type (A–F). In addition, we performed a comparison between pool of DS samples obtained from all the cells/tissues analyzed and pool of normal samples obtained from all the cells/tissues analyzed (“Total transcriptome map”, G). Detailed list of selected samples for the meta-analysis of gene expression profiles in the DS vs. normal samples for each transcriptome map has been provided in Supplementary Table S1.*

### Functional Enrichment Analysis

We have considered the biological significance of the DS/normal ratios near to 3:2 (1.5) or 2:3 (0.67) between DS and normal gene expression values due to the stimulatory or inhibitory effects, respectively, of the extra copy of Hsa21. Moreover, in order to account for natural variation in gene expression, threshold values have been arbitrarily and proportionally extended to ≥1.3 or ≤0.76, respectively. Consequently, we arbitrarily considered three intervals of ratio of the mean expression values: (1) expression ratios close to 1 (1.29–0.77), implying that the genes are not differentially expressed in DS samples; (2) expression ratios ≥1.30 and (3) expression ratios ≤0.76.

A functional enrichment analysis of over-expressed genes in the “Total transcriptome map” was performed using ‘ToppFun’ from the ‘ToppGene Suite’ Gene Ontology tool (Chen et al., 2009). We submitted a list of human genes with expression ratio ≥1.30 and a list of genes of all the chromosomes with expression ratio ≤0.76, excluding EST clusters. The selected genes were categorized according to GO classification based on their hypothetical molecular functions and biological processes. A second functional enrichment analysis of over-expressed Hsa21 genes in the “Total transcriptome map” was performed. We submitted a list of Hsa21 genes with expression ratio ≥1.30 and a list of Hsa21 genes with expression ratio ≤0.76. The analysis was assessed for Molecular Function and Biological Process categories.

### *In Vitro* Validation of the Fibroblast Transcriptome Map

In order to obtain a sample experimental confirmation of the transcriptome maps derived from the meta-analysis, we selected a group of known and characterized genes. Four groups of genes were created, according to their expression ratio A/B calculated by TRAM. The groups are the following: expression ratio A/B ≥ 2 (*BACE2, ADAMTS1*, *DHFR, DONSON*, *MX1*); expression ratio A/B between 1.4 and 1.8 (*RCAN1*, *SOD1*, *ATP5J*, *DYRK1A*); expression ratio A/B close to 1 (*ACTB*); expression ratio A/B ≤ 0.6 (*SDC2*, *SERPINF1*, *POSTN*). We also verified that selected genes were not included among the genes with a known incomplete determination of their 5′ coding sequence (Vitale et al., 2017a). We chose *GAPDH* and *B2M* as reference genes.

Primary fibroblast cell lines were collected by Galliera Genetic Bank (GGB), member of the Network Telethon of Genetic Biobanks (Baldo et al., 2016). All the cell lines were tested for mycoplasma, to exclude a possible contamination. Furthermore, a karyotype analysis was carried out by GGB to confirm the cytogenetic diagnosis. The cell lines used in this project were obtained from two non-DS donors and two DS patients. Specifically, the DS cell lines arose from a 54-year-old woman and 21-year-old man, while non-trisomic cell lines from a 50-year-old man and a 31-year-old woman. The primary cell lines, sent from GGB, had been split between 7 and 10 times.

The cells contained in the flasks were treated with the method of Chomczynski and Sacchi (1987) for total RNA extraction. The RNA quantity and quality have been verified through Nanodrop spectrophotometer (ND-1000 spectrophotometer). The reverse transcription (RT) was performed according to Caracausi et al. (2017b).

Primer pairs were designed with ‘Amplify 3’ software (Engels, 1993) following standard criteria (Sharrocks, 1994). Each primer is designed on a different exon and each primer pair binds to regions common to all splicing isoforms of the same gene since microarray probe sequences are often complementary to sequences common of the known gene isoforms and TRAM provides a unique reference value for each locus gathering all isoforms. These criteria caused a variation in the amplicon lengths between 99 and 214 bp.

Real-Time RT-PCR assays were performed in triplicate, using the CFX96 instrument (Bio-Rad Laboratories, Hercules, CA, United States). The gene expression analysis was performed using two pools of cDNA, one derived from RNA extracted from the two trisomy 21 fibroblast cell lines and the other from RNA extracted from the two non-trisomic fibroblast cell lines.

The reactions were performed in a total volume of 20 μL using Sybr Select Master Mix 2× for CFX (Applied Biosystems, by Life Technologies) according to manufacturer instructions providing the following cycling parameters: 2 min at 50°C (UDG activation), 2 min at 95°C (AmpliTaq Fast DNA Polymerase UP activation), 40 cycles of 15 s at 95°C (denature) and of 1 min at 61°C (anneal and extend). In order to assess amplification specificity, a melting step consisting of an increase in temperature of 0.5°C/s from 65°C to 95°C was performed.

For each gene we used the primer pair that gave between 90 and 110% efficiency. For the gene expression study we used the 2^-ΔΔCt^ (delta delta threshold cycle) method (Livak and Schmittgen, 2001) that calculates the expression ratio, between the trisomy 21 (test) and the euploid (control) condition of each target gene compared to one or more reference genes:

$$\begin{array}{c} 2^{-\Delta \Delta Ct}\;=\;2^{-\Delta Ct(test)\;-\;\Delta Ct(control)}\;=\\ -\;(Ct\;target\;-\;Ct\;reference)test\;-\;(Ct\;target\;-\;Ct\;reference)control \end{array}$$

Finally, we performed the bivariate statistical analysis using JMP 5.1 software (SA Institute, Campus Drive, Cary, NC, United States) between the expected ratios, generated by TRAM and the observed ratios obtained by Real-Time RT-PCR, examining their statistical correlation.

## Results

### Database Search and Database Building

The search of data related to global gene expression profile experiments in DS patients has been performed on the databases as described in “Materials and Methods” section (PubMed, GEO, and ArrayExpress) and retrieved 83 samples (for a total of 3,315,050 analyzed data points) from 10 microarray experiments on DS cells (Pool A) and 74 samples (for a total of 2,779,729 analyzed data points) from 10 microarray experiments on normal cells (Pool B). All these experiments fulfill the inclusion and exclusion criteria (see Materials and Methods section) and allow to obtain six differential transcriptome maps from brain, LCLs, blood cells, fibroblasts, thymus, and iPSCs. Moreover one “Total transcriptome map” was obtained by merging all the DS samples from any tissue as Pool A and all normal samples as Pool B. Experimental design scheme is shown in **Table 1**. Detailed list of selected samples for the meta-analysis of gene expression profiles in the Pool A (DS) and Pool B (normal) for each transcriptome map has been provided in Supplementary Table S1.

### Transcriptome Map Comparison of DS vs. Normal Samples

A first result of our analysis is a quantitative reference gene expression value for each human mapped locus after intra- and inter-sample normalization (Lenzi et al., 2011). The number of loci (from 13,167 for thymus to 37,181 for “Total transcriptome maps”) for which the comparison between the two conditions (DS vs. normal) was possible due to the presence of expression values for those loci in both sample pools considered is also provided by TRAM software.

We provide regional differential expression datasets related to all the available DS samples compared to normal samples, fulfilling inclusion and exclusion criteria, performing analyses for each cell type and a comparison between whole pool DS and whole pool normal samples (**Table 1**). Detailed results are available as Supplementary Tables S2–S8, showing a reference gene expression value for each human mapped locus in each condition analyzed. Moreover, Supplementary Tables S2–S8 showed a mean gene expression ratio between Sample A (DS) and Sample B (normal) for each locus in each comparison performed covering the whole range of the expression magnitude order as calculated by TRAM. DS/normal mean gene expression ratio ranges are 23.63–0.07 (brain, 24,699 loci analyzed); 4.31–0.03 (LCLs, 35,527 loci analyzed); 17.56–0.15 (blood, 24,699 loci analyzed); 111.51–0.02 (fibroblasts, 29,216 loci analyzed); 256.13–0.11 (thymus, 13,167 loci analyzed); 11.05–0.29 (iPSCs, 29,541 loci analyzed); 33.47–0.06 (whole pool, 37,181 loci analyzed).

**FIGURE 2:**
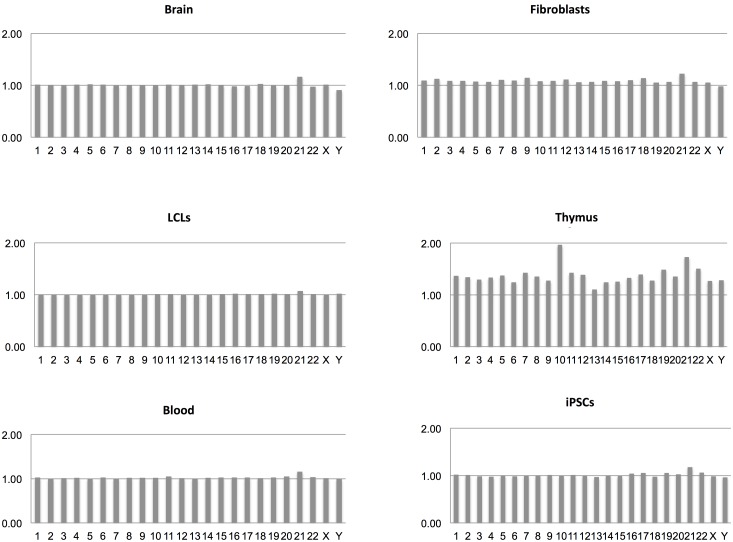
DS/normal expression ratio for each chromosome in terms of mean expression level derived from all the genes on that chromosome in each transcriptome map.

From the transcriptome maps comparing gene expression between DS and normal samples from different sources, we have obtained transcriptional frameworks useful for the identification of changes caused by the extra copy of Hsa21. Interestingly, the general pattern of gene expression across chromosomes in all the comparisons performed is very similar, always showing a prevalence of over-expressed Hsa21 genes (**Figure 2**). In particular, most of the dysregulated genes on Hsa21 reflect the 3:2 expected ratio (Supplementary Table S9). Moreover, data show that most of the DS/normal mean gene expression ratios were very close to 1, escaping gene-dosage effects whereas among the dysregulated genes the expression ratios were very near to 3:2 or 2:3 ratios, following the stimulatory or inhibitory effects, respectively, of the extra copy of Hsa21 (Supplementary Tables S2–S8).

A comparison with previous available studies is shown in Supplementary Table S10.

### Analysis of Segments in “Map” Mode

We provide the number (at least three over-/under-expressed genes) and the gene content of each genomic segment found to be statistically significantly over-/under-expressed in the comparison between the two sample sources. Each genomic segment was identified among the 12,342 segments generated and following removal of overlapping segments with similar gene content.

For each transcriptome map, we performed the analysis of segments in “Map” mode, deriving a table containing all the over-/under-expressed segments obtained from the comparison between the two pools (Supplementary Table S11).

Briefly, over-expressed segment were 8 in brain transcriptome map (6 on Hsa21), 12 in LCLs transcriptome map (9 on Hsa21), 21 in blood transcriptome map (6 on Hsa21), 8 in fibroblasts transcriptome map (1 on Hsa21), 2 in thymus transcriptome map (none on Hsa21), 5 in iPSCs transcriptome map (2 on Hsa21), 13 in “Total transcriptome map” (9 on Hsa21). Among under-expressed segments, 3 segments were found in brain, 15 in LCLs, 13 in blood, 4 in fibroblasts, 1 in thymus, 1 in iPSCs and 6 in “Total transcriptome maps”, of which none is on Hsa21.

For instance, LCLs transcriptome map shows the significant under-expression of immunoglobulin lambda variable cluster (*IGLV* on 22q11). Blood transcriptome map shows the significant over-expression of segments containing genes for hemoglobin (11p15.4 and 16p13.3). These are examples of biologically sound results consistent with known facts and obtained without any *a priori* assumption.

Overall, the analysis of segments reveals a high prevalence of Hsa21 over-expressed segments over the other genomic regions in all the transcriptome maps except thymus, suggesting, in particular, a specific region on Hsa21 that appears to be frequently over-expressed (21q22). The most frequent genes of this specific region on Hsa21 are *TMEM50B* (transmembrane protein 50B), *SON* (SON DNA binding protein) and *DONSON* (downstream neighbor of son).

### Functional Enrichment Analysis Results

The results of functional enrichment analysis, performed by ‘ToppFun’ from the ‘ToppGene Suite’ Gene Ontology tool, of over- and under-expressed genes (with expression ratios ≥1.30 and ≤0.76, respectively) in the “Total transcriptome map” (comparing all DS samples for any tissue with all normal samples), are shown in **Table 2**. Input gene lists included 1,196 and 1,228 over- and under-expressed genes resulted following exclusion of all the EST clusters (Supplementary Table S12).

**Table 2:** Results of functional enrichment analysis, performed by ‘ToppFun’ from the ‘ToppGene Suite’ Gene Ontology tool, of over- and under-expressed genes (with expression ratios ≥1.30 and ≤0.76, respectively) in the “Total transcriptome map”.

Over-expressed genes			*p*-value
**Molecular function (1/1)**			
1	GO:0019838	Growth factor binding	2.55E-05
**Biological processes (20/606)**			
1	GO:0006928	Movement of cell or subcellular component	3.03E-11
2	GO:0009790	Embryo development	1.08E-10
3	GO:0048598	Embryonic morphogenesis	1.12E-10
4	GO:0007369	Gastrulation	7.33E-10
5	GO:0060795	Cell fate commitment involved in formation of primary germ layer	1.25E-09
6	GO:0040011	Locomotion	2.32E-09
7	GO:0045165	Cell fate commitment	2.66E-09
8	GO:0071363	Cellular response to growth factor stimulus	2.70E-09
9	GO:0000904	Cell morphogenesis involved in differentiation	2.75E-09
10	GO:0009719	Response to endogenous stimulus	2.82E-09
11	GO:0016477	Cell migration	2.87E-09
12	GO:0070848	Response to growth factor	3.14E-09
13	GO:0007498	Mesoderm development	5.22E-09
14	GO:0060322	Head development	6.76E-09
15	GO:0022008	Neurogenesis	6.83E-09
16	GO:0048699	Generation of neurons	7.99E-09
17	GO:0040007	Growth	1.35E-08
18	GO:0071495	Cellular response to endogenous stimulus	1.41E-08
19	GO:0009887	Animal organ morphogenesis	1.46E-08
20	GO:0030182	Neuron differentiation	1.63E-08

**Under-expressed genes**			***p*-value**

**Molecular function (20/47)**			
1	GO:0003823	Antigen binding	6.39E-15
2	GO:0005102	Receptor binding	2.31E-11
3	GO:0005201	Extracellular matrix structural constituent	1.21E-10
4	GO:0005539	Glycosaminoglycan binding	2.76E-10
5	GO:0005178	Integrin binding	8.40E-09
6	GO:0008201	Heparin binding	1.29E-07
7	GO:0050839	Cell adhesion molecule binding	1.33E-07
8	GO:0019838	Growth factor binding	2.30E-07
9	GO:0005518	Collagen binding	2.70E-07
10	GO:0030881	Beta-2-microglobulin binding	4.12E-07
11	GO:0005125	Cytokine activity	6.00E-07
12	GO:1901681	Sulfur compound binding	9.46E-07
13	GO:0034987	Immunoglobulin receptor binding	1.31E-06
14	GO:0046983	Protein dimerization activity	2.41E-06
15	GO:0004252	Serine-type endopeptidase activity	7.61E-06
16	GO:0005509	Calcium ion binding	1.08E-05
17	GO:0048407	Platelet-derived growth factor binding	1.92E-05
18	GO:0008236	Serine-type peptidase activity	2.83E-05
19	GO:0017171	Serine hydrolase activity	3.58E-05
20	GO:0030883	Endogenous lipid antigen binding	4.40E-05
**Biological processes (20/822)**			***p*-value**
1	GO:0002682	Regulation of immune system process	4.23E-26
2	GO:0006955	Immune response	1.55E-25
3	GO:0007155	Cell adhesion	8.55E-21
4	GO:0022610	Biological adhesion	1.96E-20
5	GO:0006952	Defense response	6.56E-20
6	GO:0002250	Adaptive immune response	1.23E-19
7	GO:0050776	Regulation of immune response	2.59E-19
8	GO:0030198	Extracellular matrix organization	4.25E-19
9	GO:0043062	Extracellular structure organization	4.97E-19
10	GO:0002684	Positive regulation of immune system process	1.26E-18
11	GO:0001775	Cell activation	2.54E-17
12	GO:0045321	Leukocyte activation	1.12E-16
13	GO:0046649	Lymphocyte activation	6.67E-16
14	GO:0071345	Cellular response to cytokine stimulus	2.68E-15
15	GO:0002252	Immune effector process	5.26E-15
16	GO:0006959	Humoral immune response	5.98E-15
17	GO:0050778	Positive regulation of immune response	1.08E-14
18	GO:0002460	Adaptive immune response based on somatic recombination of immune receptors built from immunoglobulin superfamily domains	1.85E-14
19	GO:0019221	Cytokine-mediated signaling pathway	1.95E-14
20	GO:0045087	Innate immune response	2.35E-14

Among over-expressed genes, the prevailing and significant processes concern embryogenesis, cell growth and neurogenesis. Among under-expressed genes, immune system processes prevail.

The results of functional enrichment analysis of the subset of the over- and under-expressed genes (with expression ratios ≥1.30 and ≤0.76, respectively) located on Hsa21 are shown in **Table 3**. Input gene lists included 95 and 9 over- and under-expressed genes resulted following exclusion of all the EST clusters (Supplementary Table S13). Regarding genes with expression ratio ≥1.30, nitrite reductase activity, cystathionine beta-synthase activity, hydroxymethyl-, formyl- and related transferase activity, oxidoreductase activity are among the significant molecular functions (involving the following genes: *CBSL*, *CBS*, *SLC19A1*, *GART*, and *FTCD*). The most significant biological processes concern cysteine and homoserine metabolisms.

**Table 3:** Results of functional enrichment analysis, performed by ‘ToppFun’ from the ‘ToppGene Suite’ Gene Ontology tool, of over- and under-expressed genes (with expression ratio ≥1.30 and ≤0.76, respectively) located on Hsa21 in the “Total transcriptome map”.

Over-expressed genes			*p*-value
**Molecular function (11/11)**			
1	GO:0050421	Nitrite reductase (NO-forming) activity	1.78E-05
2	GO:0004122	Cystathionine beta-synthase activity	1.78E-05
3	GO:1904047	*S*-Adenosyl-L-methionine binding	1.78E-05
4	GO:0004124	Cysteine synthase activity	1.78E-05
5	GO:0098809	Nitrite reductase activity	1.78E-05
6	GO:0070025	Carbon monoxide binding	1.78E-05
7	GO:0070026	Nitric oxide binding	5.29E-05
8	GO:0072341	Modified amino acid binding	2.73E-04
9	GO:0016742	Hydroxymethyl-, formyl- and related transferase activity	4.87E-04
10	GO:0016662	Oxidoreductase activity, acting on other nitrogenous compounds as donors, cytochrome as acceptor	6.25E-04
11	GO:0016661	Oxidoreductase activity, acting on other nitrogenous compounds as donors	1.14E-03
**Biological processes (6/6)**			
1	GO:0006535	Cysteine biosynthetic process from serine	1.44E-05
2	GO:0019343	Cysteine biosynthetic process via cystathionine	4.29E-05
3	GO:0019344	Cysteine biosynthetic process	8.56E-05
4	GO:0009092	Homoserine metabolic process	1.42E-04
5	GO:0070814	Hydrogen sulfide biosynthetic process	1.42E-04
6	GO:0019346	Transsulfuration	1.42E-04

**Under-expressed genes**			***p*-value**

**Molecular function (2/2)**			
1	GO:0005212	Structural constituent of eye lens	3.69E-03
2	GO:0051082	Unfolded protein binding	1.65E-02

### TRAM Result Validation by Real-Time RT-PCR

To validate the results of the meta-analysis performed by TRAM software, experiments of Real-Time RT-PCR were conducted, following criteria described in the “Materials and Methods” section. The primer pairs used are listed in Supplementary Table S14.

**FIGURE 3:**
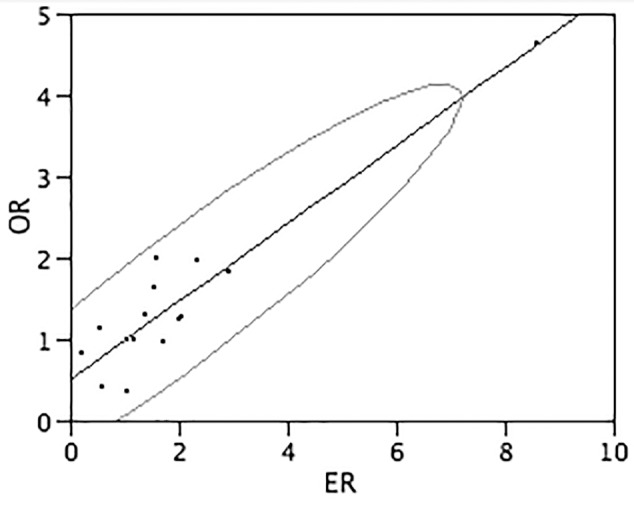
Correlation between observed end expected gene expression ratios. The graph shows the correlation between the gene expression ratios observed (*Y*-axis) and expected (*X*-axis) calculated by bivariate analysis by JMP 5.1 program. Assuming that the variables have a bivariate normal distribution, the ellipse (Bivariate Normal Ellipse) contains about 95% of the points. The narrowness of the ellipse shows the correlation of the variables. If the ellipse is narrow and diagonally oriented, the variables are related. Pearson correlation coefficient = 0.93 and *p*-value < 0.0001.

The gene expression ratios observed by Real-Time RT-PCR between DS and normal conditions and mean features obtained from *in vitro* and *in silico* analyses, for each target gene, are shown numerically in **Table 4** and graphically in **Figure 3**. The correlation between the observed and expected gene expression ratios, performed by bivariate analysis using JMP 5.1 program, is statistically highly significant (Pearson correlation coefficient = 0.93 and *p*-value < 0.0001) (**Figure 3**).

**Table 4:** Genes selected for the validation *in vitro* of the transcriptome map of fibroblasts by Real-Time RT-PCR.

Gene symbol	EEV A	EEV B	ER	OR
*RCAN1*	453.79	263.66	1.72	0.98
*SDC2*	437.68	823.53	0.53	1.14
*SERPINF1*	194.90	328.31	0.59	0.41
*SOD1*	2,937.37	1,845.43	1.59	2.00
*POSTN*	373.55	1,866.22	0.20	0.82
*BACE2*	191.80	95.41	2.01	1.25
*ACTB*	5,048.13	4,775.04	1.06	0.37
*ADAMTS1*	1,132.72	552.20	2.05	1.27
*ATP5J*	1,459.89	944.98	1.54	1.64
*DHFR*	151.07	51.51	2.93	1.84
*DONSON*	107.93	45.93	2.35	1.96
*DYRK1A*	120.45	87.71	1.37	1.30
*MX1*	446.86	52.12	8.57	4.65

*From left to right: official symbol of the gene; expected expression value (EEV) of each gene in Pool A and Pool B calculated by TRAM; expected ratio (ER) between DS and non-DS fibroblasts provided by TRAM; observed ratio (OR) determined by Real-Time RT-PCR with the method of 2^-ΔΔCt^ provided by Bio-Rad CFX Manager Software 2.1.*

## Discussion

The study of differences in gene expression among DS and control individuals has become one of the central issues of DS research. The presence of an extra Hsa21 inevitably leads to an altered expression of genes within it, but it has not yet been clarified how this alteration leads to the onset of the typical symptoms in DS. The annotation of the long arm of Hsa21 sequence in 2000 (Hattori et al., 2000) allowed to identify, to date, 273 validated or reviewed known genes on Hsa21 (Piovesan et al., 2016), which have become the objects of various research aimed to assess their expression in DS and their involvement in pathways and molecular mechanisms that may be related to the pathogenesis of DS (Vilardell et al., 2011; Briggs et al., 2013; Weick et al., 2013; Letourneau et al., 2014; Olmos-Serrano et al., 2016; Sullivan et al., 2016).

To date, meta-analyses about DS gene expression have been performed by Vilardell et al. (2011) and Guedj et al. (2016), both choosing to integrate human and murine data. Moreover Vilardell et al. (2011) analyzed data obtained through different quantitative (microarray, Real-Time RT-PCR, MALDI) and qualitative techniques (SAGE, Western Blot). The meta-analysis approach is a very useful and effective method for summarizing data from several studies, leading to a higher statistical power and more significant conclusions than those drawn on the basis of individual studies. In particular, these meta-analyses showed that the majority of dysregulated genes were not located on Hsa21, although, proportionally, Hsa21 contains the greater number of over-expressed genes, suggesting an important role of trisomic genes in the global gene expression alteration (Vilardell et al., 2011) and the largest number of differentially expressed genes mapped to the 21q11-21q22.3 chromosomal location (Guedj et al., 2016). However, these studies based their meta-analysis approach on scoring the recurrence of a result across multiple reports.

Our original approach is instead able to integrate previous data at numerical level, generating quantitative maps including expression level provided as a consensus, reference value for each gene analyzed in at least one experiment. This in turn allows the determination of DS/normal ratio of expression for any gene, along with identification of differentially expressed genomic segments based on quantitative measure of the RNA output of the segment rather than a simple enrichment in differentially expressed genes within the segment. Another unique feature of our quantitative mapping approach is the inclusion of uncharacterized loci such as EST clusters. The study of these sequences, whose functions are still unknown, might be useful to identify new transcripts related to the pathogenesis of trisomy 21, representing a potential new field of investigation for future studies.

Together with all the described advantages, a disadvantage we can identify in this type of analysis in comparison to the elaboration of the gene expression profiles presented in the original reports releasing the datasets used by us is the additional work needed to perform the analysis, including manual critical curation to identify suitable samples, uniformation of the data which may be presented as linear numbers, natural logarithm (ln) or binary logarithm (log_2_*n*) of the raw spot intensity level, and set up by feeding the TRAM software with updated human genomic data before starting the analysis with the desired parameters. In addition, an intrinsic limitation of microarray-based expression profile datasets is the not comprehensive coverage of human genes due to incomplete representation of all the relative probes on the analysis platforms (for example, for the most representative platform in our analysis, GPL570, we calculated the coverage of 84% of the whole 22,451 currently known human genes and of 93% of the 18,255 protein-coding gene subset) (Piovesan et al., 2016). Finally, it should be noted that protein expression levels might be different from the mRNA abundance due to post-transcription regulation (Liu et al., 2017).

Following systematic selection of all the available DS vs. normal microarray experiments from different tissues and data integration allowed by uniform probe-to-gene assignment as well as intra- and inter-sample normalization as described in “Materials and Methods” section, we obtained a systematic, quantitative database of gene expression in different tissues useful for comparing DS and normal tissues.

We decided to select only human samples, excluding samples derived from mice because of the incomplete human trisomy 21 in the context of mouse models (Weick et al., 2013).

Samples derived from fetal or embryonic tissues/annexes were not considered in our analysis because of the small amount of available data related to this condition. Moreover, concerning gene expression patterns, information about the origin of embryonic tissues/annexes (regular pregnancy, spontaneous or induced ending of pregnancy) might be relevant because complications or miscarriages often occur due to genetic alterations causing gene expression changes. Further studies including embryonic gene expression patterns might be useful for understanding critical changes during development.

Although gene expression is normally affected by a gender bias (Shah et al., 2014), pathological alterations due to a clear effect of an autosome are not expected to be related to gender-biased manifestations, in accordance with the fact that no main clinical difference has been reported between males and females in DS (Tolksdorf and Wiedemann, 1981) as well as with the recent demonstration of DS-specific alterations in metabolome irrespectively of gender (Caracausi et al., 2018). However, we provide the gender for each sample included in our analysis (Supplementary Table S1) in order to allow further analysis regarding this aspect.

The TRAM database allows to search every single gene of interest and to observe the corresponding expression ratio between DS and normal samples, the expression values for each biological condition and for each sample in the two pools and the number of data points and samples used for the analysis.

In the standardized tables we provide, one can test any hypothesis regarding general or specific alterations of gene expression due to extra copy of Hsa21. For instance, in the case of the *SOD1* gene for which a 3:2 gene dosage effect has been well known for decades, also for protein product, the reference expression values expressed as percentage of the mean value (DS/normal) are: brain, 1,807/1,236 (ratio 1.46); LCLs, 1,762/1,259 (1.40); blood, 1,467/1,138 (1.29); fibroblasts, 2,937/1,845 (1.59); thymus, 751/488 (1.54); iPSCs, 1,774/1,701 (1.04), thus providing evidence of differences in expression levels of *SOD1* in different tissues, as well as excellent across-tissue conservation of the 1.5:1 ratio expected from the additional Hsa21. Interestingly, iPSCs biological model appears farther from the primary tissues under this aspect. A modest increase of *SOD1* expression in DS iPSCs compared to normal cells was also found by Weick et al. (2013).

The analysis performed by TRAM yield results regarding expression patterns at chromosome level and at single gene level; moreover, TRAM allows to generate quantitative gene expression data which can be used for further studies, e.g., functional analyses.

Regarding whole chromosomes, the graphs representing the DS/normal expression ratio for each chromosome (in terms of mean expression level derived from all the genes on that chromosome) showed that the most over-expressed chromosome, in proportion, is Hsa21 in all analyzed maps, in accordance with the most recently published results (Weick et al., 2013; Olmos-Serrano et al., 2016; Sullivan et al., 2016). These data indicate that, although a fraction of Hsa21 genes escapes dosage effects, Hsa21 genes are selectively over-expressed in DS samples compared to genes on other chromosomes, reflecting a decisive role of the extra Hsa21 in the pathogenesis of the syndrome.

Regarding individual genes, the effect of an extra copy of Hsa21 on the cellular transcriptome remains an open issue in understanding the pathogenesis of DS.

Interestingly, analyzing all the expression ratios obtained for each comparison, most of the DS/normal ratios were very close to 1, escaping gene-dosage effects whereas among the dysregulated genes the expression ratios were very near to 3:2 or 2:3. These observations are consistent with the hypothesis that (1) the presence of an extra copy of Hsa21 resulted in increments in the transcriptional activity of Hsa21 and (2) the downstream effects of trisomy 21 might reflect the enhancer or silencer activity of Hsa21 genes on other genes in the genome for which the gene expression values result in 150% or 67%, respectively.

In each transcriptome map the genes with extreme profiles are on other chromosomes than Hsa21, consistent with the hypothesis that 3:2 gene dosage effects have their origin on Hsa21 and the chain of effects may propagate throughout the genome amplifying the final effect on specific genes. For example, *JAKMIP3*, located on chromosome 10, (ratio 256.13 in thymus transcriptome map) is the Janus kinase and microtubule interacting protein 3 and has been found expressed at highest levels in the central nervous system and in endocrine tissues (Cruz-Garcia et al., 2007) and is thought to contribute in the maintenance of TrkA-mediated nerve growth factor (NGF) signaling in neurons (Diaz-Ruiz et al., 2013). *BEX5*, located on chromosome X, belongs to the brain-expressed X-linked family which is known to play a role in neuronal development (Alvarez et al., 2005) while in our brain transcriptome map it is under-expressed in DS (ratio 0.07). Furthermore, non-coding RNAs (*ZNF667-AS1* over-expressed in brain and iPSCs and *H19* over-expressed in brain transcriptome maps) and unmapped loci are found among extreme profile genes, pointing to the need of further investigations on the DS pathogenesis.

Among the most commonly Hsa21 over-expressed genes, *TMEM50B* gene has been identified as a candidate for DS brain phenotypes by Lein et al. (2007) and was found over-expressed in human adult and re-analyzed fetal DS brain (Lockstone et al., 2007) datasets and in mouse cerebellum of DS models (Moldrich et al., 2008).

Regarding chromosome segments, the analysis showed that a significant number of over-expressed segments belongs to Hsa21 in all transcriptome maps. The originality of TRAM software consists in determining the expression value of each segment not depending on the number of genes included in the segment but measuring the mean of the expression values of the genes included in that segment. This parameter is indicative of the actual transcription level of that specific DNA region, removing the bias deriving from the number of genes contained in it. Interestingly, in the brain map, six of the eight significantly over-expressed segments result on Hsa21. In particular, the most represented over-expressed region maps on the long arm of Hsa21, specifically on 21q22 band in all the analyzed tissues (except thymus). The 21q22 band includes the HR-DSCR (located on 21q22.13) (Pelleri et al., 2016), although its expression cannot be evaluated due to the absence of probes in the considered platforms of currently known genes in the HR-DSCR.

An interesting possibility is to generate trisomy 21 cells with the selective deletion of a single copy of the HR-DSCR through CRISPR/Cas 9 system (Bauer et al., 2015). Through this approach it would be possible to perform functional studies on this region followed by gene expression analyses. This might yield biological insights about new regulative pathways involved in DS pathogenesis.

Comparing genomic segments that we found significantly over-/under-expressed in DS vs. normal fibroblasts transcriptome map with dysregulated domains in fetal skin primary fibroblasts derived from the study by Letourneau et al. (2014), some discrepancies could be due to the methodological and biological differences (array vs. RNA-seq, 3.2 Mb vs. 500 Kb size and adult vs. fetus samples), but the over-expression of chromosomes 10, 18, and 21 segments is confirmed.

We performed a functional enrichment study of over- and under-expressed genes in all the genome and Hsa21 over- and under-expressed genes. In particular, the over-expressed gene analyses highlighted molecular and biological mechanisms involving cell development that may be related to several characteristic features of trisomy 21 and are consistent with previous studies (Lockstone et al., 2007; Weick et al., 2013). Enriched biological processes resulted from the analysis concern the embryogenesis, cell growth and neurogenesis. These processes represent the main alterations that have been correlated to the ID of trisomy 21. DS subjects have a reduced head circumference, brachycephaly, cerebral atrophy and abnormalities in the cerebral cortex, brain stem, and cerebellum (Pinter et al., 2001). In DS brains, there is a general reduction of cortex development and an anomalous formation and localization of neurons (Guidi et al., 2011). Also dendritic arborization is affected, limiting contacts between neurons and other cells (Becker et al., 1986). The embryo development reflects a general alteration that turns into congenital anomalies and malformations that occur during prenatal life of DS subjects, such as heart and gastrointestinal defects, skeletal anomalies and many others. Moreover, the functional enrichment analysis of Hsa21 genes with expression ratio ≥ 1.30 showed that the most enriched Hsa21 molecular functions involving the *CBS*, *GART* and *FTCD* gene products might be related to the one carbon cycle including the folic acid cycle and the homocysteine pathway. These data are coherent with the general outlook of metabolic disturbances leading to mental retardation performed by Prof. J. Lejeune, who stated: “As a very broad and very tentative hypothesis, it could be postulated that in case of mental retardation in which there is no gross anatomic defect of the brain, no obvious disturbance of the insulating substances, no demonstrated abnormality of the membranes building blocks, a deficiency of the one carbon cycle could be the most likely trouble to be looked for” (Lejeune, 1981).

To test the reliability of the transcriptome maps generated by TRAM software, we chose to validate the fibroblast transcriptome map performing an experimental validation of the obtained data on DS and normal fibroblast cell lines. The very good correlation coefficient (*r* = 0.93, *p*-value < 0.0001) between the values obtained by meta-analysis of multiple datasets and independent samples assayed by Real-Time RT-PCR, despite the high biological variability of the samples and the limits deriving from the comparison between two different methods, demonstrates the high reliability of TRAM results.

Our study suggests that a specific region of Hsa21 (21q22) might contain most sensitive over-expressed genes involved in DS pathogenesis and that a complex interaction between trisomic genes and other dysregulated regions of the genome could exist and not only a direct correlation of Hsa21 genes with DS symptoms. Several mechanisms such as negative feedback, dosage compensation or epigenetic gene expression variation could explain this apparent discordance between the genomic dosage imbalance and the expression levels of Hsa21 genes (Antonarakis, 2001, 2017; Prandini et al., 2007). Surely, it would be useful to determine which mechanisms control the expression pattern of Hsa21 genes and furthermore whether non-trisomic gene deregulation is stochastic or if it is the result of the influence of Hsa21 genes on specific non-Hsa21 genes. The identification of the mechanisms at the basis of the expression of these genes remains one of the crucial points of DS research in order to characterize molecular pathways and molecular targets for targeted drug treatments.

This work could be extended to a higher number of samples by adding more types of tissues or cells and also including RNA-seq data, a high-throughput method that has been spreading in the last years and could contribute significantly to the addition of more and relevant data, although to date a very minor number of datasets, obtained through this method, is available for DS. Our datasets provide a standardized, quantitative reference model useful for further studies of transcription in DS.

## Author Contributions

MCP designed the study, collected the data, and performed the analysis. CC and MC contributed to the analysis of the data and performed the experimental validation. LV, FA, PS, and AP contributed to the analysis of the data. MCP, CC, and PS wrote the manuscript draft. GC and CL contributed to the discussion of the data. AP and MC supervised the project. All authors read, critically discussed, and approved the final manuscript.

## Conflict of Interest Statement

The authors declare that the research was conducted in the absence of any commercial or financial relationships that could be construed as a potential conflict of interest.

## Footnotes

1 https://www.ncbi.nlm.nih.gov/pubmed

2 http://www.ebi.ac.uk/arrayexpress/

3 http://apollo11.isto.unibo.it/software
