# Supplementary material for: Integrated Quantitative Transcriptome Maps of Human Trisomy 21 Tissues and Cells
Source: Front Genet. 2018 Apr 24;9:125. doi: 10.3389/fgene.2018.00125 (PMC5928158; doi:10.3389/fgene.2018.00125)
Supplement: Supplementary file 1 [file Table_1.DOCX]

**Integrated quantitative transcriptome maps of human trisomy 21 tissues and cells**

Maria Chiara Pelleri, Chiara Cattani, Lorenza Vitale, Francesca Antonaros, Pierluigi Strippoli, Chiara Locatelli, Guido Cocchi, Allison Piovesan* and Maria Caracausi

***To whom correspondence should be addressed.** Tel: +39 0512094113; Fax: +39 0512094110; e-mail address: allison.piovesan2@unibo.it

**Supplementary Table S1.** List of selected samples for the meta-analysis of gene expression profiles in the DS (Down syndrome) Pool A and normal Pool B for each transcriptome map. A) brain; B) lymphoblastoid cell lines, LCLs; C) blood; D) fibroblasts; E) thymus; F) induced pluripotent stem cells, iPSCs. From left to right: ID (identification code) of the sample in the study; experiment ID; platform name; sample ID; sex of sex of sample donor (NA: not available); source of the sample; platform type; number of spots of the platform and bibliographic reference.

| **A) TRAM DS Brain vs. normal Brain** | | | | | | | | |
| --- | --- | --- | --- | --- | --- | --- | --- | --- |
| **Pool A DS Brain (n=13)** | | | | | | | | |
| **ID** | **Series** | **Platform ID** | **Sample** | **Sex** | **Source** | **Array-Platform title** | **Sample Rows** | **Reference** |
| A1-A7 | GSE5390 | GPL96 | GSM123264  GSM123265  GSM123266  GSM123267  GSM123268  GSM123269  GSM123270 | F  F  F  M  F  M  M | DS prefrontal cortex | [HG-U133A] Affymetrix Human Genome U133A Array | 22283 | Lockstone et al. 2007 |
| A8-A13 | GSE48611 | GPL570 | GSM1182334-39 | M | Ts21 neurons biological replicate 1-2-3 in duplicate | [HG-U133_Plus_2] Affymetrix Human Genome U133 Plus 2.0 Array | 54675 | Weick et al. 2013 |
| **Pool B normal Brain (n=11)** | | | | | | | | |
| B1-B8 | GSE5390 | GPL96 | GSM123271 GSM123272  GSM123273  GSM123274  GSM123275  GSM123276  GSM123277  GSM123278 | M  F  M  M  M  M  M  M | Healthy control brain | [HG-U133A] Affymetrix Human Genome U133A Array | 22283 | Lockstone et al. 2007 |
| B9-B11 | GSE48611 | GPL570 | GSM1182340-42 | M | Euploid neurons biological replicate 1-2-3 in duplicate | [HG-U133_Plus_2] Affymetrix Human Genome U133 Plus 2.0 Array | 54675 | Weick et al. 2013 |

| **B) TRAM DS LCL vs. normal LCLs** | | | | | | | | |
| --- | --- | --- | --- | --- | --- | --- | --- | --- |
| **Pool A DS LCL (n=17)** | | | | | | | | |
| **ID** | **Series** | **Platform ID** | **Sample** | **Sex** | **Source** | **Array-Platform title** | **Sample Rows** | **Reference** |
| A1-A11 | GSE34458 | GPL6947 | GSM849493  GSM849495  GSM849497  GSM849499  GSM849501  GSM849503  GSM849505  GSM849507  GSM849509  GSM849511 GSM849514 | F  M  M  M  M  M  M  F  F  M  F | Lymphoblastoid cell line Trisomic 21 CHD replicate 1-11 | Illumina HumanHT-12 V3.0 expression beadchip | 48803 | Ripoll et al. 2012 |
| A12-17 | E-MTAB-1238 | A-AFFY-44  =GPL570 | 36  37  38  45  56  130 | F  M  F  M  F  M | LCLs | [HG-U133_Plus_2] | 54675 | Granese et al. 2013 |
| **Pool B normal LCLs (n=18)** | | | | | | | | |
| B1-B12 | GSE34458 | GPL6947 | GSM849494  GSM849496  GSM849498  GSM849500  GSM849502  GSM849504  GSM849506  GSM849508 GSM849510  GSM849512 GSM849513  GSM849515 | F  F  M  M  F  M  F  M  F  M  M  F | Lymphoblastoid cell line Euploid replicate 1-12 | Illumina HumanHT-12 V3.0 expression beadchip | 48803 | Ripoll et al. 2012 |
| B13-B18 | E-MTAB-1238 | A-AFFY-44  =GPL570 | CT1  CT2  CT27  CT31  CT37  CT39 | F  M  F  M  M  F | LCLs | [HG-U133_Plus_2] | 54675 | Granese et al. 2013 |

| **C) TRAM DS Blood vs. normal Blood** | | | | | | | | | |
| --- | --- | --- | --- | --- | --- | --- | --- | --- | --- |
| **Pool A DS Blood cells (n=6)** | | | | | | | | | |
| **ID** | **Series** | | **Platform ID** | **Sample** | **Sex** | **Source** | **Array-Platform title** | **Sample Rows** | **Reference** |
| A1-A6 | Sent by authors | | GPL570 | DG1088 DG1218 DG2698 DG3077 DG3197 DG3563 Down | 2M  4F | Peripheral blood leucocytes | [HG-U133_Plus_2] Affymetrix Human Genome U133 Plus 2.0 Array | 54675 | Salemi et al. 2012 |
| **Pool B normal Blood Cells (n=5)** | | | | | | | | | |
| B1-B5 | | Sent by authors | GPL570 | DG1163 DG1301 DG1755 DG3933 DG4076 Normal | 2M  3F | Peripheral blood leucocytes | [HG-U133_Plus_2] Affymetrix Human Genome U133 Plus 2.0 Array | 54675 | Salemi et al. 2012 |

| **D) TRAM DS Fibroblasts vs. normal Fibroblasts** | | | | | | | | |
| --- | --- | --- | --- | --- | --- | --- | --- | --- |
| **Pool A DS Skin Fibroblasts (n=11)** | | | | | | | | |
| **ID** | **Series** | **Platform ID** | **Sample** | **Sex** | **Source** | **Array-Platform title** | **Sample Rows** | **Reference** |
| A1-A5 | GSE9762 | GPL570 | GSM246324-26, 29-30 | NA | TS21 skin fibroblasts | [HG-U133_Plus_2] Affymetrix Human Genome U133 Plus 2.0 Array | 54675 | Pevsner unpublished |
| A6-A11 | GSE42956 | GPL10558 | GSM1053958-60, 82-84 | M | DS fibroblast CCL54 replicates 1-6 | Illumina HumanHT-12 V4.0 expression beadchip | 47309 | Briggs et al. 2013 |
| **Pool B normal Skin Fibroblasts (n=14)** | | | | | | | | |
| B1-B5 | GSE9762 | GPL570 | GSM246321-23, 27-28 | NA | Euploid skin fibroblasts | [HG-U133_Plus_2] Affymetrix Human Genome U133 Plus 2.0 Array | 54675 | Pevsner unpublished |
| B6-B14 | GSE42956 | GPL10558 | GSM1053955-57, 61-63, 76-78 | M | WT fibroblast CRL-2429 replicates 1-8 | Illumina HumanHT-12 V4.0 expression beadchip | 47309 | Briggs et al. 2013 |

| **E) TRAM DS Thymus vs. normal Thymus** | | | | | | | | |
| --- | --- | --- | --- | --- | --- | --- | --- | --- |
| **Pool A DS Thymus (n=4)** | | | | | | | | |
| **ID** | **Series** | **Platform ID** | **Sample** | **Sex** | **Source** | **Array-Platform title** | **Sample Rows** | **Reference** |
| A1-A4 | GSE23910 | GPL6480 | GSM589475  GSM589488  GSM589489  GSM589490 | F  M  F  F | DS thymus | Agilent-014850 Whole Human Genome Microarray 4x44K G4112F | 21940 | Lima et al. 2011 |
| **Pool B normal Thymus (n=4)** | | | | | | | | |
| B1-B4 | GSE23910 | GPL6480 | GSM589449  GSM589467  GSM589470  GSM589491 | M  M  M  F | Control thymus | Agilent-014850 Whole Human Genome Microarray 4x44K G4112F | 21940 | Lima et al. 2011 |

| **F) TRAM DS iPSCs vs. normal iPSCs** | | | | | | | | |
| --- | --- | --- | --- | --- | --- | --- | --- | --- |
| **Pool A DS iPSCs (n=32)** | | | | | | | | |
| **ID** | **Series** | **Platform ID** | **Sample** | **Sex** | **Source** | **Array-Platform title** | **Sample Rows** | **Reference** |
| A1-A2 | GSE38931 | GPL10558 | GSM952367, 70 | F | Trisomic fibroblast-derived iPSC | Illumina HumanHT-12 V4.0 expression beadchip | 47323 | Li et al. 2012 |
| A3-A14 | GSE42956 | GPL10558 | GSM1053964-66, 70-72, 91-96 | M | DS fibroblast-derived iPSC  CCL54 replicate 1-6 in duplicate | Illumina HumanHT-12 V4.0 expression beadchip | 47309 | Briggs et al. 2013 |
| A15-20 | GSE48611 | GPL570 | GSM1182325-30 | M | Ts21 iPSC biological replicate 1-3 in duplicate | [HG-U133_Plus_2] Affymetrix Human Genome U133 Plus 2.0 Array | 54675 | Weick et al. 2013 |
| A21-32 | GSE47014 | GPL15207 | GSM1142933-35, 39-41, 45-47, 51-53 | F | DS-iPS:  Parental Replicate 1-3  Clone 1 Replicate 1-3  Clone 2 Replicate 1-3  Clone 3 Replicate 1-3 | [PrimeView] Affymetrix Human Gene Expression Array | 49293 | Jiang et al. 2013 |
| **Pool B normal IPSCs (n=22)** | | | | | | | | |
| B1-B4 | GSE38931 | GPL10558 | GSM952368-69,71-72 | F | Disomic iPSC derived from trisomic cells in duplicate | Illumina HumanHT-12 V4.0 expression beadchip | 47323 | Li et al. 2012 |
| B5-B16 | GSE42956 | GPL10558 | GSM1053943-45, GSM1053967-69, GSM1053973-75, GSM1053988-90 | M  M  M  F | WT-fibroblast-derived iPSC in triplicate;  Euploid DS-iPSC in triplicate;  WT- normal iPSC. | Illumina HumanHT-12 V4.0 expression beadchip | 47309 | Briggs et al. 2013 |
| B17-B19 | GSE48611 | GPL570 | GSM1182331-33 | M | Euploid iPSC biological replicate 1-3 | [HG-U133_Plus_2] Affymetrix Human Genome U133 Plus 2.0 Array | 54675 | Weick et al. 2013 |
| B20-B22 | GSE47014 | GPL15207 | GSM1142930-32 | M | Male iPS Replicate 1-3 | [PrimeView] Affymetrix Human Gene Expression Array | 49293 | Jiang et al. 2013 |
