# Supplementary material for: Integrated Quantitative Transcriptome Maps of Human Trisomy 21 Tissues and Cells
Source: Front Genet. 2018 Apr 24;9:125. doi: 10.3389/fgene.2018.00125 (PMC5928158; doi:10.3389/fgene.2018.00125)
Supplement: Supplementary file 11 [file Table_11.DOCX]

**Integrated quantitative transcriptome maps of human trisomy 21 tissues and cells**

Maria Chiara Pelleri, Chiara Cattani, Lorenza Vitale, Francesca Antonaros, Pierluigi Strippoli, Chiara Locatelli, Guido Cocchi, Allison Piovesan* and Maria Caracausi

***To whom correspondence should be addressed**. Tel: +39 0512094113; Fax: +39 0512094110; e-mail address: allison.piovesan2@unibo.it

**Supplementary Table S11**. Genomic segments significantly over-/under-expressed in each transcriptome map (in order: A) brain; B) LCLs; C) blood; D) fibroblasts; E) thymus; F) iPSCs; G) total) resulted by "Map" mode analysis. Segments are sorted by decreasing expression ratio. In the "Map" mode, TRAM displays UniGene EST clusters (with the prefix "Hs." in the case of *Homo sapiens*) only if they have an expression value. For simplicity, some segments are not shown because they overlap with those highlighted in one of the listed regions. Chr: chromosome; Location: segment cytoband derived from that of the first mapped gene within the segment; Segment Start/End: chromosomal coordinates for each segment; Genes in the segment: over-expressed gene in red; under-expressed gene in blue.

| **A) TRAM DS Brain vs. NL Brain** | | | | | | |
| --- | --- | --- | --- | --- | --- | --- |
| **Chr** | **Location** | **Segment Start** | **Segment End** | **Value DS/normal** | **q-value** | **Genes in the segment** |
| **Over-expressed segments** | | | | | | |
| chr19 | 19q13.43 | 56,000,001 | 56,500,000 | 3.82 | 0.000497785 | LINC01864 ZNF667 ZNF667-AS1 Hs.732277 |
| chr1 | 1p31.1 | 74,250,001 | 74,750,000 | 2.37 | 0.000548672 | ERICH3 ERICH3-AS1 CRYZ TYW3 |
| chr21 | 21q22.2 | 39,000,001 | 39,500,000 | 1.60 | 0.000140113 | PSMG1 HMGN1 WRB SH3BGR |
| chr21 | 21q22.11 | 31,500,001 | 32,000,000 | 1.50 | 0.000693596 | LOC102724449 SCAF4 HUNK |
| chr21 | 21q21.1 | 17,500,001 | 18,000,000 | 1.49 | 0.000538462 | CXADR BTG3 C21orf91 |
| chr21 | 21q11.2 | 14,000,001 | 14,500,000 | 1.48 | 0.000538462 | LIPI HSPA13 SAMSN1 |
| chr21 | 21q22.11 | 33,250,001 | 33,750,000 | 1.46 | 0.000634748 | IL10RB TMEM50B SON DONSON |
| chr21 | 21q21.3 | 25,500,001 | 26,000,000 | 1.44 | 0.001282752 | Hs.388313 MRPL39 JAM2 GABPA |
| **Under-expressed segments** | | | | | | |
| chr14 | 14q32.2 | 100,750,001 | 101,250,000 | 0.63 | 0.000504433 | MEG3 Hs.710763 SNORD114-3 |
| chr11 | 11p15.4 | 4,750,001 | 5,250,000 | 0.58 | 0.00044056 | HBB HBD HBG1 |
| chrX | Xq22.1 | 101,750,001 | 102,250,000 | 0.58 | 0.000448914 | ZMAT1 TCEAL2 BEX5 |

| **B) TRAM DS LCLs vs. NL LCLs** | | | | | | |
| --- | --- | --- | --- | --- | --- | --- |
| **Chr** | **Location** | **Segment Start** | **Segment End** | **Value DS/normal** | **q-value** | **Genes in the segment** |
| **Over-expressed segments** | | | | | | |
| chr21 | 21q22.3 | 43,750,001 | 44,250,000 | 1.25 | 3.89574E-10 | PDXK CSTB RRP1 AATBC AGPAT3 PWP2 C21orf33 ICOSLG |
| chr21 | 21q11.2 | 14,500,001 | 15,000,000 | 1.22 | 0.000417554 | SAMSN1 SAMSN1-AS1 NRIP1 |
| chr21 | 21q22.11 | 34,000,001 | 34,500,000 | 1.19 | 0.000900824 | MRPS6 SMIM11A LOC105372793 |
| chr21 | 21q22.11 | 33,250,001 | 33,750,000 | 1.19 | 2.01431E-06 | IL10RB TMEM50B GART DONSON CRYZL1 Hs.655910 |
| chr21 | 21q22.11 | 33,500,001 | 34,000,000 | 1.18 | 1.50505E-06 | GART DONSON CRYZL1 Hs.655910 ATP5O LINC00649 |
| chr21 | 21q22.3 | 44,750,001 | 45,250,000 | 1.18 | 4.22123E-06 | UBE2G2 SUMO3 PTTG1IP ITGB2 ITGB2-AS1 FAM207A |
| chr21 | 21q22.3 | 46,250,001 | 46,750,000 | 1.16 | 0.000123032 | MCM3AP YBEY PCNT PRMT2 |
| chr17 | 17p13.3 | 1 | 500,000 | 1.15 | 0.002352367 | Hs.113418 Hs.461807 RFLNB |
| chr21 | 21q22.13 | 36,750,001 | 37,250,000 | 1.15 | 5.01288E-05 | Hs.580941 LOC105372795 PIGP TTC3 DSCR3 |
| chr21 | 21q21.2 | 25,250,001 | 25,750,000 | 1.15 | 1.34128E-05 | LINC00158 MIR155HG LINC00515 MRPL39 JAM2 ATP5J |
| chrX | Xq22 | 103,000,001 | 103,500,000 | 1.13 | 0.006845787 | BEX2 TCEAL9 BEX3 |
| chr16 | 16q13 | 56,250,001 | 56,750,000 | 1.13 | 1.67828E-05 | GNAO1 MT2A MT1E MT1G MT1H MT1X |
| **Under-expressed segments** | | | | | | |
| chr2 | 2p11.2 | 88,500,001 | 89,000,000 | 0.90 | 0.003696948 | EIF2AK3 IGKC Hs.742004 |
| chr11 | 11q23.3 | 118,000,001 | 118,500,000 | 0.90 | 0.001129263 | IL10RA JAML CD3D CD3G |
| chr4 | 4q13.3 | 75,000,001 | 75,500,000 | 0.90 | 0.000560014 | PARM1 LINC02562 RCHY1 |
| chr12 | 12p13.2 | 10,250,001 | 10,750,000 | 0.89 | 9.43569E-06 | KLRD1 LOC101928100 KLRC4-KLRK1 KLRK1 KLRC4 KLRC2 Hs.554559 |
| chr4 | 4q13.3 | 73,500,001 | 74,000,000 | 0.89 | 0.003058322 | RASSF6 CXCL8 PF4V1 |
| chr11 | 11q14.1 | 85,750,001 | 86,250,000 | 0.89 | 0.001389513 | SYTL2 PICALM Hs.658368 |
| chr22 | 22q11.22 | 22,500,001 | 23,000,000 | 0.88 | 0.000550063 | LL22NC03-63E9.3 IGLV4-3 IGLL5 IGLC1 IGLJ3 |
| chr17 | 17q23.1 | 59,750,001 | 60,250,000 | 0.87 | 0.001389513 | MIR21 RPS6KB1 Hs.221899 |
| chr22 | 22q11.22 | 22,000,001 | 22,500,000 | 0.86 | 0.000389259 | IGLV4-60 IGLV6-57 VPREB1 IGLV1-44 |
| chr14 | 14q32.33 | 106,750,001 | 107,250,000 | 0.85 | 0.001389513 | IGH Hs.443491 IGHV3-73 |
| chr7 | 7q36.1 | 150,250,001 | 150,750,000 | 0.84 | 2.84248E-06 | REPIN1 LINC00996 GIMAP7 GIMAP4 GIMAP6 GIMAP1-GIMAP5 GIMAP5 |
| chr2 | 2p11.2 | 88,750,001 | 89,250,000 | 0.81 | 0.001389513 | IGKC Hs.742004 IGKV1-17 |
| chr1 | 1q25.1 | 174,250,001 | 174,750,000 | 0.77 | 0.000232606 | RABGAP1L Hs.673577 Hs.659569 |
| chr7 | 7p14.1 | 38,000,001 | 38,500,000 | 0.77 | 0.000560014 | TARP TRGC2 TRGV9 AMPH |
| chr15 | 15q26.2 | 94,000,001 | 94,500,000 | 0.75 | 9.87658E-05 | LINC01581 MCTP2 Hs.664781 |

| **C)TRAM DS Blood vs. NL Blood** | | | | | | |
| --- | --- | --- | --- | --- | --- | --- |
| **Chr** | **Location** | **Segment Start** | **Segment End** | **Value DS/normal** | **q-value** | **Genes** |
| **Over-expressed segments** | | | | | | |
| chr11 | 11p15.4 | 5,000,001 | 5,500,000 | 2.59 | 0.001182588 | HBB HBD HBG1 HBG2 |
| chr14 | 14q12 | 24,500,001 | 25,000,000 | 2.42 | 0.000498793 | CTSG GZMH GZMB |
| chr1 | 1q32.1 | 203,000,001 | 203,500,000 | 2.42 | 0.003609812 | PPFIA4 CHI3L1 CHIT1 |
| chr3 | 3p21.31 | 46,000,001 | 46,500,000 | 2.04 | 0.001250702 | CCR3 CCR5 LTF |
| chr19 | 19p13.3 | 500,001 | 1,000,000 | 1.55 | 0.02456603 | AZU1 PRTN3 ELANE |
| chr4 | 4q13.3 | 73,750,001 | 74,250,000 | 1.47 | 0.000188812 | PF4V1 CXCL1 PF4 CXCL5 CXCL2 |
| chr6 | 6p22.3 | 16,250,001 | 16,750,000 | 1.44 | 0.000228719 | GMPR ATXN1 Hs.663425 |
| chr1 | 1p36.12 | 22,250,001 | 22,750,000 | 1.44 | 0.000890176 | C1QA C1QC C1QB |
| chr11 | 11q25 | 134,250,001 | 134,750,000 | 1.43 | 0.000890176 | Hs.657530 GLB1L2 B3GAT1 |
| chr7 | 7p14.1 | 38,000,001 | 38,500,000 | 1.42 | 0.000734266 | TARP TRGC2 TRGV9 |
| chr21 | 21q22.3 | 46,000,001 | 46,500,000 | 1.40 | 2.9635E-06 | COL6A2 LSS MCM3AP-AS1 MCM3AP PCNT DIP2A |
| chr21 | 21q22.3 | 46,250,001 | 46,750,000 | 1.38 | 7.57349E-06 | MCM3AP-AS1 MCM3AP PCNT DIP2A PRMT2 |
| chr21 | 21q22.3 | 45,750,001 | 46,250,000 | 1.34 | 1.63448E-05 | Hs.721038 COL6A2 LSS MCM3AP-AS1 MCM3AP |
| chr12 | 12p13.31-p13.2 | 10,000,001 | 10,500,000 | 1.31 | 0.005311795 | CLEC12B OLR1 KLRD1 KLRC4 |
| chr16 | 16p13.3 | 1 | 500,000 | 1.30 | 0.015833801 | HBM HBA1 HBQ1 |
| chr21 | 21q22.3 | 43,750,001 | 44,250,000 | 1.30 | 1.63448E-05 | PDXK AATBC AGPAT3 TRAPPC10 C21orf33 |
| chr21 | 21q22.3 | 43,500,001 | 44,000,000 | 1.29 | 0.000149821 | RRP1B PDXK AATBC AGPAT3 |
| chr2 | 2q35 | 217,750,001 | 218,250,000 | 1.28 | 0.000890176 | TNS1 CXCR2 CXCR1 |
| chr10 | 10q23.31 | 89,000,001 | 89,500,000 | 1.27 | 0.001749208 | IFIT2 IFIT3 IFIT1 |
| chr20 | 20q11.21 | 31,250,001 | 31,750,000 | 1.26 | 0.003609812 | ID1 BCL2L1 TPX2 |
| chr21 | 21q22.11 | 33,250,001 | 33,750,000 | 1.26 | 0.000692453 | TMEM50B GART SON DONSON |
| **Under-expressed segments** | | | | | | |
| chr19 | 19q13.41 | 53,000,001 | 53,500,000 | 0.83 | 1.30758E-05 | ZNF415 ZNF347 ZNF665 ZNF677 ZNF813 |
| chr19 | 19p13.2 | 11,750,001 | 12,250,000 | 0.82 | 9.96789E-07 | ZNF441 ZNF439 Hs.658583 ZNF763 ZNF844 ZNF136 Hs.715702 |
| chr12 | 12p11.21 | 31,500,001 | 32,000,000 | 0.81 | 0.000352448 | DENND5B AMN1 Hs.668507 |
| chr9 | 9q32 | 112,500,001 | 113,000,000 | 0.78 | 0.000534106 | Hs.657664 SLC46A2 ZNF883 |
| chr2 | 2p25.3 | 1 | 500,000 | 0.77 | 0.000244862 | FAM110C SH3YL1 ALKAL2 |
| chr4 | 4p14 | 38,750,001 | 39,250,000 | 0.76 | 0.001166138 | TLR10 Hs.659564 TMEM156 |
| chr2 | 2q11.2 | 99,500,001 | 100,000,000 | 0.74 | 0.000244862 | AFF3 Hs.663031 Hs.656220 |
| chr2 | 2p16.1 | 60,500,001 | 61,000,000 | 0.74 | 0.000244862 | BCL11A Hs.735341 REL |
| chr2 | 2q24.3 | 164,750,001 | 165,250,000 | 0.70 | 1.70466E-05 | COBLL1 Hs.136017 SLC38A11 SCN3A |
| chr12 | 12q21.33 | 92,000,001 | 92,500,000 | 0.67 | 0.000352448 | LINC01619 LINC02397 Hs.554101 |
| chr1 | 1q23.1 | 157,500,001 | 158,000,000 | 0.64 | 5.73188E-05 | FCRL5 FCRL3 FCRL2 FCRL1 |
| chr6 | 6q15 | 90,000,001 | 90,500,000 | 0.58 | 3.99543E-05 | BACH2 Hs.665002 Hs.660736 |
| chr20 | 20p12.1 | 15,750,001 | 16,250,000 | 0.50 | 3.99543E-05 | MACROD2 Hs.549963 LOC613266 |

| **D) TRAM DS Fibroblasts vs. NL Fibroblasts** | | | | | | |
| --- | --- | --- | --- | --- | --- | --- |
| **Chr** | **Location*** | **Segment Start** | **Segment End** | **Value DS/normal** | **q-value** | **Genes in the segment** |
| **Over-expressed segments** | | | | | | |
| chr10 | 10q11.23-q21.1 | 52,000,001 | 52,500,000 | 2.46 | 0.000588784 | DKK1 Hs.61596 LINC01468 |
| chr21 | 21q22.2 | 41,000,001 | 41,500,000 | 2.42 | 0.000758397 | BACE2 MX2 MX1 |
| chr7 | 7p15.2 | 26,750,001 | 27,250,000 | 2.04 | 1.70295E-05 | HOTAIRM1 HOXA7 HOXA9 HOXA9 HOXA10-AS HOXA11 Hs.662980 |
| chr10 | 10q21.3 | 68,000,001 | 68,500,000 | 2.04 | 0.001638726 | HERC4 MYPN PBLD |
| chr17 | 17q21.2 | 41,500,001 | 42,000,000 | 2.03 | 0.001638483 | KRT19 KRT14 KRT16 KRT17 JUP |
| chr17 | 17q21.2 | 41,000,001 | 41,500,000 | 2.01 | 1.14337E-05 | KRTAP1-5 KRTAP1-3 KRTAP1-1 KRTAP2-1 KRTAP9-8 KRTAP9-4 KRT33B KRT34 |
| chr18 | 18q21.33 | 63,750,001 | 64,250,000 | 1.93 | 0.001058562 | SERPINB7 SERPINB2 HMSD LINC01924 |
| chr10 | 10q23.33 | 94,250,001 | 94,750,000 | 1.87 | 0.000758397 | PLCE1 HELLS Hs.658935 |
| **Under-expressed segments** | | | | | | |
| chr7 | 7p15.1 | 28,500,001 | 29,000,000 | 0.63 | 0.000392523 | CREB5 TRIL CPVL |
| chr20 | 20p12.2 | 10,500,001 | 11,000,000 | 0.63 | 0.000505598 | Hs.224012 JAG1 Hs.664262 |
| chr6 | 6q23.2 | 134,000,001 | 134,500,000 | 0.57 | 0.000122274 | SLC2A12 SGK1 LOC101928231 |
| chr3 | 3q24 | 148,500,001 | 149,000,000 | 0.41 | 0.000122274 | AGTR1 CPB1 CPA3 |

| **E) TRAM DS Thymus vs. NL Thymus** | | | | | | |
| --- | --- | --- | --- | --- | --- | --- |
| **Chr** | **Location** | **Segment Start** | **Segment End** | **Value DS/normal** | **q-value** | **Genes in the segment** |
| **Over-expressed segments** | | | | | | |
| chr19 | 19p13.3 | 1 | 500,000 | 6.20 | 0.000788904 | THEG SHC2 MADCAM1 |
| chrX | Xq28 | 152,500,001 | 153,000,000 | 4.43 | 0.000297994 | CSAG2 CSAG1 MAGEA6 |
| **Under-expressed segments** | | | | | | |
| chr4 | 4q25 | 107,750,001 | 108,250,000 | 0.40 | 6.07254E-05 | CYP2U1 HADH LEF1 |

| **F) TRAM DS iPSCs vs. NL iPSCs** | | | | | | |
| --- | --- | --- | --- | --- | --- | --- |
| **Chr** | **Location*** | **Segment Start** | **Segmen tEnd** | **Value DS/normal** | **q-value** | **Genes in the segment** |
| **Over-expressed segments** | | | | | | |
| chr11 | 11p15.5 | 2,000,001 | 2,500,000 | 1.77 | 0.008134395 | H19 IGF2 TH |
| chr21 | 21q22.3 | 44,500,001 | 45,000,000 | 1.61 | 4.32E-13 | KRTAP10-1 KRTAP10-5 KRTAP10-8 KRTAP10-9 KRTAP10-10 KRTAP10-11 KRTAP12-3 KRTAP12-2 KRTAP12-1 KRTAP10-12 LINC01424 Hs.734565 LINC01547 |
| chr21 | 21q22.3 | 44,000,001 | 44,500,000 | 1.48 | 0.004913277 | PWP2 DNMT3L Hs.655733 |
| chr2 | 2q21.1 | 129,750,001 | 130,250,000 | 1.43 | 0.001865892 | RAB6C MZT2B TUBA3E |
| chr2 | 2q32.1 | 186,500,001 | 187,000,000 | 1.39 | 0.001345232 | Hs.697073 Hs.656287 FAM171B |
| **Under-expressed segments** | | | | | | |
| chr2 | 2q31.1 | 172,750,001 | 173,250,000 | 0.61 | 0.000299299 | RAPGEF4 Hs.674047 MAP3K20-AS1 |

| **G) Total Transcriptome Map** | | | | | | |
| --- | --- | --- | --- | --- | --- | --- |
| **Chr** | **Location** | **Segment Start** | **Segment End** | **Value DS/normal** | **q-value** | **Genes in the segment** |
| **Over-expressed segments** | | | | | | |
| chr21 | 21q22.3 | 44,250,001 | 44,750,000 | 1.43 | 1.51752E-10 | DNMT3L Hs.655733 KRTAP10-1 KRTAP10-3 KRTAP10-5 KRTAP10-8 KRTAP10-10 KRTAP12-3 KRTAP12-2 KRTAP12-1 KRTAP10-12 |
| chr21 | 21q22.3 | 43,750,001 | 44,250,000 | 1.43 | 0.000416628 | CSTB RRP1 PWP2 DNMT3L |
| chr21 | 21q22.3 | 44,500,001 | 45,000,000 | 1.42 | 4.7388E-11 | KRTAP10-1 KRTAP10-3 KRTAP10-5 KRTAP10-8 KRTAP10-10 KRTAP12-3 KRTAP12-2 KRTAP12-1 KRTAP10-12 UBE2G2 ITGB2-AS1 LINC01547 |
| chr21 | 21q22.3 | 42,750,001 | 43,250,000 | 1.40 | 1.96439E-05 | PDE9A NDUFV3 ERVH48-1 U2AF1 CRYAA |
| chr21 | 21q22.3 | 43,500,001 | 44,000,000 | 1.34 | 0.002188333 | RRP1B CSTB RRP1 |
| chr10 | 10q23.31 | 89,750,001 | 90,250,000 | 1.32 | 0.000734935 | Hs.557901 Hs.538383 Hs.538381 |
| chr10 | 10q11.23-q21.1 | 52,250,001 | 52,750,000 | 1.29 | 0.002727315 | DKK1 Hs.61596 Hs.380362 Hs.553319 |
| chr21 | 21q22.11 | 33,750,001 | 34,250,000 | 1.27 | 0.00394097 | ITSN1 LINC00649 SLC5A3 |
| chr21 | 21q22.3 | 43,000,001 | 43,500,000 | 1.25 | 0.004255006 | U2AF1 CRYAA SIK1 |
| chr7 | 7p14.1 | 38,000,001 | 38,500,000 | 1.25 | 0.001081885 | TARP TRGC2 TRGV9 |
| chr21 | 21q22.3 | 46,000,001 | 46,500,000 | 1.24 | 0.00605622 | SPATC1L LSS Hs.596577 |
| chr21 | 21q22.11 | 33,250,001 | 33,750,000 | 1.24 | 0.004314369 | SON DONSON ITSN1 |
| chr11 | 11p15.5 | 2,000,001 | 2,500,000 | 1.22 | 0.011177958 | H19 IGF2 TH |
| **Under-expressed segments** | | | | | | |
| chr14 | 14q32.33 | 106,250,001 | 106,750,000 | 0.80 | 0.001636389 | IGH Hs.553354 IGHV3-48 |
| chr1 | 1q23.1 | 157,500,001 | 158,000,000 | 0.79 | 0.000150155 | FCRL5 FCRL3 FCRL2 FCRL1 |
| chr4 | 4q25 | 107,750,001 | 108,250,000 | 0.78 | 0.000757319 | SGMS2 CYP2U1 LEF1 |
| chr2 | 2p11.2 | 88,750,001 | 89,250,000 | 0.78 | 0.000216388 | IGKC Hs.742004 IGKV1-5 IGKV1-17 |
| chr9 | 9q34.3 | 134,750,001 | 135,250,000 | 0.77 | 0.001636389 | COL5A1 OLFM1 Hs.583991 |
| chr3 | 3q24 | 148,500,001 | 149,000,000 | 0.75 | 0.000587948 | AGTR1 CPB1 CPA3 |
