# Supplementary material for: Integrated Quantitative Transcriptome Maps of Human Trisomy 21 Tissues and Cells
Source: Front Genet. 2018 Apr 24;9:125. doi: 10.3389/fgene.2018.00125 (PMC5928158; doi:10.3389/fgene.2018.00125)
Supplement: Supplementary file 14 [file Table_14.DOCX]

**Integrated quantitative transcriptome maps of human trisomy 21 tissues and cells**

Maria Chiara Pelleri, Chiara Cattani, Lorenza Vitale, Francesca Antonaros, Pierluigi Strippoli, Chiara Locatelli, Guido Cocchi, Allison Piovesan* and Maria Caracausi

***To whom correspondence should be addressed.** Tel: +39 0512094113; Fax: +39 0512094110; e-mail address: allison.piovesan2@unibo.it

**Supplementary Table S14.** List of genes used for in vitro validation of the fibroblast transcriptome map indicating GenBank accession number of the RNA sequence, the sequences of the primers pairs used (top: forward primer; bottom: reverse primer) and the lenght of the amplification products.

| Gene symbol | Gene full name | GenBank accession number of RNA sequence | Primers pair sequence  (5´→ 3´) | RT-PCR  product  (bp) |
| --- | --- | --- | --- | --- |
| *RCAN1* | regulator of calcineurin 1 | NM_004414 | ctggagcttcattgactgcgag gtgatgtccttgtcatacgtcc | **153** |
| *SDC2* | syndecan 2 | NM_002998.3 | aggagtgtatcctattgatgacg gtttcttcaggtgactttgtctg | **198** |
| *SERPINF1* | serpin family F member 1 | NM_002615 | ggctgtctccaacttcggcta cggtgaatgatggattctgttcg | **150** |
| *SOD1* | superoxide dismutase 1, soluble | NM_000454 | tagcgagttatggcgacgaag ggtacagcctgctgtattatctc | **186** |
| *POSTN* | periostin | NM_001135934 | ttggctcatagtcgtatcaggg cattctcatataaccagggcaac | **174** |
| *BACE2* | beta-site APP-cleaving enzyme 2 | NM_012105 | ggtcttacttccctaaaatctcc gaaatgccgaatcggtaacattc | **142** |
| *ACTB* | actin beta | NM_001101.3 | gagcatcccccaaagttcacaat cttcctgtaacaacgcatctcata | **99** |
| *B2M* | beta-2-microglobulin | NM_004048.2 | ctactctctctttctggcctgg cccagacacatagcaattcagg | **114** |
| *GAPDH* | glyceraldehyde-3-phosphate dehydrogenase | NM_002046 | caacgaccactttgtcaagc ctgtgaggaggggagattca | **214** |
| *ADAMTS1* | ADAM metallopeptidase with thrombospondin type 1 motif 1 | NM_006988 | gtcatccacgatgaacagaagg tcagccatcccaagagtatcac | **191** |
| *ATP5J* | ATP synthase, H+ transporting, mitochondrial Fo complex subunit F6 | NM_001685 | tcttcagaggctcttcaggttc tactctgaactagcatcaacagg | **195** |
| *DHFR* | dihydrofolate reductase | NM_000791 | agaagacctggttctccattcc aactgccaccaactatccagac | **195** |
| *DONSON* | downstream neighbor of SON | NM_017613 | ttaaaacgcgactccttttcacc gagccaatagataaggctctgc | **191** |
| *DYRK1A* | dual specificity tyrosine phosphorylation regulated kinase 1A | NM_001396 | attcagagtcgcttttatcggtc ggtcaagaatatgagcaggtgg | **196** |
| *MX1* | MX dynamin like GTPase 1 | NM_001144925 | gaacgaagataagtggagaggc gaacgaagataagtggagaggc | **143** |
